# Genetic Differences in Dorsal Hippocampus Acetylcholinesterase Activity Predict Contextual Fear Learning Across Inbred Mouse Strains

**DOI:** 10.3389/fpsyt.2021.737897

**Published:** 2021-10-18

**Authors:** Sean M. Mooney-Leber, Dana Zeid, Prescilla Garcia-Trevizo, Laurel R. Seemiller, Molly A. Bogue, Stephen C. Grubb, Gary Peltz, Thomas J. Gould

**Affiliations:** ^1^Department of Psychology, University of Wisconsin-Stevens Point, Stevens Point, WI, United States; ^2^Department of Biobehavioral Health, The Pennsylvania State University, State College, PA, United States; ^3^The Jackson Laboratory, Bar Harbor, ME, United States; ^4^Department of Anesthesiology, Perioperative and Pain Medicine, Stanford University, Palo Alto, CA, United States

**Keywords:** hippocampus, learning, acetylcholinesterase, genetics, fear conditioning

## Abstract

Learning is a critical behavioral process that is influenced by many neurobiological systems. We and others have reported that acetylcholinergic signaling plays a vital role in learning capabilities, and it is especially important for contextual fear learning. Since cholinergic signaling is affected by genetic background, we examined the genetic relationship between activity levels of acetylcholinesterase (AChE), the primary enzyme involved in the acetylcholine metabolism, and learning using a panel of 20 inbred mouse strains. We measured conditioned fear behavior and AChE activity in the dorsal hippocampus, ventral hippocampus, and cerebellum. Acetylcholinesterase activity varied among inbred mouse strains in all three brain regions, and there were significant inter-strain differences in contextual and cued fear conditioning. There was an inverse correlation between fear conditioning outcomes and AChE levels in the dorsal hippocampus. In contrast, the ventral hippocampus and cerebellum AChE levels were not correlated with fear conditioning outcomes. These findings strengthen the link between acetylcholine activity in the dorsal hippocampus and learning, and they also support the premise that the dorsal hippocampus and ventral hippocampus are functionally discrete.

## Introduction

Learning is a complex behavioral process that relies on multiple neurobiological systems working in concert. The cholinergic system is one such system whose signaling modulates learning and memory networks (1, 2). For example, the acetylcholine muscarinic receptor (mAChR) antagonist scopolamine has been shown to impair learning in a contextual fear conditioning task (3, 4), spatial learning in the Morris Water Maze (5, 6), passive avoidance learning (6, 7), and object recognition learning (8). Other work has found that signaling via nicotinic acetylcholine receptors (nAChR) may modulate learning and memory.

Signaling via nAChR systems can enhance learning through interactions with other neurotransmitter systems. nAChR antagonism with mecamylamine, a non-selective nAChR ligand, does not impair fear conditioning (9, 10). However, mecamylamine paired with a subthreshold dose of an NMDA glutamate receptor antagonist disrupts fear conditioning (10). NMDA receptor signaling acts upstream of synaptic plasticity mediating several forms of learning and memory [for review see (11)]. Results of NMDA receptor and nAChR co-antagonism suggest that these two systems mediate similar learning-related processes, with the nAChR system perhaps subordinate to NMDA receptor signaling. In support, several studies have found that the administration of nicotine (a nAChR agonist) enhances learning (9, 12–14). In addition, nicotine reversed NMDA receptor antagonism-induced deficits in fear conditioning, and direct drug infusion experiments revealed that the dorsal hippocampus mediated this effect (15). Other studies have found that antagonism of nAChR receptors with mecamylamine alone was sufficient to impair learning (6, 16). Importantly, both of these studies used larger mecamylamine doses, and other work suggests that mecamylamine may act as an NMDA receptor antagonist at higher doses (17–19). Thus, impairment of learning via mecamylamine at higher doses may represent its influence on NMDA receptors directly instead of or in addition to actions at nAChRs. In sum, the cholinergic system is involved in learning, with the muscarinic subsystem directly mediating learning-associated cell signaling and the nicotinic system potentially interacting with glutamatergic signaling cascades to modulate learning.

Acetylcholine, the endogenous ligand of mAChRs and nAChRs, is primarily synthesized at axon terminals from choline and acetyl coenzyme A by choline acetyltransferase (ChAT). Acetylcholinesterase (AChE) works to rapidly metabolize acetylcholine into acetate and choline in the synaptic space (20). Manipulation of both acetylcholine synthesis and metabolism can alter learning (21, 22). Prevention of acetylcholine metabolism via AChE inhibition has been used to mitigate cognitive impairments associated with many neurodegenerative diseases (23–25).

A growing body of literature suggests that genetic variability in AChE-related genes could influence learning. Single nucleotide polymorphisms (SNP) in the *ACHE* gene, which encodes the acetylcholinesterase enzyme, have been identified in humans (26, 27). Valle et al. (28) reported that heritable variations in the *ACHE* gene may underlie individual differences in AChE expression and secretion. Genotype at a SNP found in the *ACHE* gene also predicted responsivity to cognitive-enhancing drug treatment in patients with dementia (29). Genetic variation impacting cholinergic signaling and associated cognition has also been reported in outbred and inbred rodent models (30, 31). Matson et al. (32) found significant differences in AChE activity in the brain cortex and red blood cells between 8 inbred mouse strains. Further, Schwegler et al. (33) reported that the density of cholinergic fibers within the hippocampus varied systematically by genetic background, which correlated with learning outcomes in two spatial learning tasks. Further evidence supporting the role of AChE in learning has been documented elsewhere (34). These findings support that the presence of genetic variants related to cholinergic systems may have a measurable impact on learning outcomes.

Inbred mouse strains provide a powerful tool for identifying the genetic contributions to various behavioral outcomes since each inbred strain has a fixed homozygous genome. Fear conditioning is a simple form of learning that is often utilized to study cognitive performance in rodent models. Studies using large inbred mouse strain panels have demonstrated that learning capabilities in components of fear conditioning are, in part, driven by genetic background (35–37). Neuroanatomical contributors to fear conditioning have been identified (38). Specifically, the dorsal hippocampus is the primary processer of contextual information during fear conditioning (39), but it is not critically involved in cued fear conditioning (40). These neuroanatomical divisions can be leveraged to assess hippocampus-dependent and -independent learning in a fear conditioning model. Since cholinergic signaling within the hippocampus is vital for fear learning (4), genetic variation in acetylcholine signaling could contribute to inter-strain variation in fear conditioning.

Therefore, we examine genetic variability in AChE activity in three brain regions (dorsal hippocampus, ventral hippocampus, and cerebellum) in 20 inbred mouse strains. The dorsal and ventral hippocampus were separately examined due to their distinct roles in fear conditioning. Specifically, the dorsal hippocampus is primarily involved in cognitive processing, whereas the ventral hippocampus regulates stress and the emotional response to fear (41). The cerebellum was selected as a control due to its lack of participation in contextual or cued fear conditioning (42). Contextual and cued fear conditioning were used to understand how genetic variation in AChE activity correlated with strain differences in hippocampus-dependent learning. We hypothesized that learning capabilities and brain AChE activity would vary significantly between strains; and because of the prominent role that the hippocampus plays in fear learning, we predicted that hippocampal AChE activity levels would be correlated with fear conditioning.

## Methods

### Subjects

Male 129S1/SvlmJ, 129S4/SvJaeJ, 129S8/SvEvNimrJ, A/J, AKR/J, BALB/cJ, BTBRT < +>ltpr3 < tf>/J, C3H/HeJ, C57BL/6J, CBA/J, DBA/1J, DBA/2J, FVB/NJ, LP/J, MA/MyJ, NZB/BINJ, SJL/J, SM/J, & SWR/J mice were obtained from Jackson Laboratory (Bar Harbor, ME). 129S2/SvPasCrl were obtained from Charles River (Wilmington, MA). Individual strain characteristics can be found on https://mice.jax.org/ and https://www.criver.com/ (129S2). These mice were part of a larger project examining the influence of genetic background on sensitivity to drugs of abuse. All mice were 10–15 weeks of age for behavioral testing and tissue collection (*n* = 9–13 per strain). All mice were group-housed [with the exception of SJL/J, which were single-housed due to excessive social aggression characteristic of this strain; (43)] with a 12-h light/dark cycle and unlimited access to food and water. All behavioral testing occurred between 8:00 A.M. and 5:00 P.M. All procedures were conducted in accordance with the NIH Guide for the Care and Use of Laboratory Animals and approved by the Penn State University IACUC Committee.

### Saline Exposure

As mentioned previously, mice from this study served as saline controls for a larger project examining the impact of nicotine on learning and memory. As a result of their experimental assignment, mice used for the current study were exposed to chronic saline for 12 days. Saline was administered via subcutaneous osmotic minipumps (model #1002, Alzet Inc.; Cupertino, CA, USA). Surgical implantation and removal of the minipumps were performed under 3.5% isoflurane anesthesia using aseptic procedures. Pumps were removed 1 day prior to behavioral training.

### Apparatus

Fear conditioning training and testing for contextual fear learning occurred in four identical noise-attenuating chambers with metal bar grid flooring (18.8 × 20 × 18.3 cm, MED Associates, St. Albans, VT, USA). Testing for cued fear learning was conducted in a separate room in four identical noise-attenuating chambers (20.32 × 22.86 × 17.78 cm, MED Associates, St. Albans, VT, USA) designed to have distinct sensory cues (different chamber size, solid plastic flooring, background vanilla odor) to allow subjects to distinguish them from the training/context test chambers. Both sets of chambers were equipped with side-mounted speakers for cued stimuli presentation (85 dB white noise) and fans to provide ventilation and background noise (65 dB). Freezing behavior was recorded using cameras mounted to chamber ceilings (Ikegami, Tokyo, Japan) connected to Noldus media recording software (Noldus, Wageningen, Netherlands). Stimuli presentation during training and both testing sessions was controlled by Med-PC software and hardware (MED Associates, St. Albans, VT, USA).

### Fear Conditioning

Mice were trained and tested in both contextual and cued fear conditioning, as previously established in our laboratory (37). Briefly, for training, mice were placed in an operant chamber for a total of 5 min. The first 2 min of training consisted of a stimulus-free period (baseline) followed by 2 conditioned stimulus (CS; 30-s 85 dB white noise)—unconditioned stimulus (US; 2-s 0.45 mA foot shock) pairings presented 2 min apart, in which the US overlapped with the last 2-s of the CS. The 2-min period in between the two CS-US pairings served as the immediate or post-shock period. Two associations are formed during the training trial and are used to assess fear conditioning: (1) Between the tone CS and footshock US (cued fear conditioning) and (2) Between the footshock US and the context/environment (contextual fear conditioning). To assess the strength of these unique forms of learning and memory, mice were tested for both contextual and cued learning 24 h after training. To test contextual fear learning, mice were placed back inside the training chamber over a 5-min trial with no stimulus presentation. Cued testing occurred at least 1 h after context testing, for which mice were placed in a novel chamber for a 3-min baseline assessment (pre-cue), followed by 3-min of CS exposure (cued). Chambers used for context and cued testing were not counterbalanced to be consistent with previous studies examining the impact of nicotine withdrawal on fear learning (37) and because contextual fear learning was the primary focus on this study. Novel cues (plastic flooring and vanilla scent) were present during cued testing to minimize generalization to the training chambers. All sessions were video recorded. Freezing behavior was tracked during all sessions via EthoVision XT (Noldus, Wageningen, Netherlands).

### Tissue Collection and AChE Activity

Twenty-four hours following the fear conditioning test, mice were sacrificed for the collection of hippocampus and whole cerebellum. Hippocampi were further dissected into dorsal and ventral sections (1:1 ratio). All tissue was flash frozen on dry ice and stored at −80°C until further processing. Brain tissue from 5 to 6 mice was randomly selected from each strain. Tissue was homogenized in 1X RIPA buffer solution (R0278, Sigma Life Sciences, St. Louis, MO, USA) with HALT Protease and Phosphatase Inhibitor Cocktail at a ratio of 100:1 (78445, Fisher Scientific, Pittsburgh, PA, USA). Homogenates were spun down at 14,000 g at 4°C for 30 min. Total protein concentration of the supernatant was determined by DC Protein Assay (500-0112; Bio-Rad, Hercules, CA, USA). Each supernatant sample was further diluted in RIPA buffer to a standard 30 μg total protein. AChE activity in diluted samples was measured using the Abcam Acetylcholinesterase Assay Kit (ab138871, Abcam, Cambridge, MA, USA) per manufacturer instructions. Inter- and intra-assay duplicate CV% were all <15%.

### Mouse Phenome Database Gene Polymorphism Queries

To assess sequence variation in genes encoding AChE and other proteins relevant to cholinergic signaling, genes were surveyed using the Mouse Phenome Database SNP data retrieval tool (44). SNP and indel data were retrieved from the Sanger4 (45) data set because of its extensive genome coverage. However, only a subset of 13 strains was analyzed (DBA/1J, C57BL/6J, LP/J, BALB/cJ, DBA/2J, 129S1/SvImJ, CBA/J, C3H/HeJ, A/J, AKR/J, NZB/BINJ, FVB/NJ, BTBR T+ Itpr3tf/J) due to the other strains not being represented in the Sanger4 data set. These data were compiled with gene function and length data from Mouse Genome Informatics website [http://www.informatics.jax.org/index.shtml (June, 2021)] in Supplementary File 1.

### Heritability Estimates and Behavioral Correlations

Heritability estimates were based on within- and between-strain variance produced from one-way ANOVAs for each behavioral and biological outcome. Briefly, sum of squares between strains (between strain variance) was divided by the summation of sum of squares between and within-strains [within strain variance; (46)]. To explore potential genetic overlap between the collected behavioral and biological variables, strain mean Pearson r correlations between all fear conditioning and AChE activity variables were computed. Pearson r correlations were also computed between AChE activity strain means and other behavioral phenotypes available through a custom dataset within Mouse Phenome Database [fear conditioning (Mooney1) and AChE data (Mooney2) collected for this study are available at https://phenome.jax.org]. A significance threshold of *p* < 0.01 (based on Pearson *r*) was applied to correlations calculated for AChE activity strain means in each brain region. As we were specifically interested in behavioral outcomes related to AChE activity, correlations significant at *p* < 0.01 derived from behavioral assays are highlighted (see Supplementary Tables 1–3).

### Statistical Analysis

Separate one-way ANOVAs with strain as a between-subjects factor were utilized to examine conditioning variables (baseline, immediate, context, pre-cue, & cued freezing) and brain AChE activity levels (dorsal hippocampus, ventral hippocampus, and cerebellum). ANOVAs with a significant effect of strain were followed up with Tukey HSD *post-hoc*. If Levene's test of homogeneity of variance was violated, a Games-Howell *post-hoc* was conducted instead. Associations between fear conditioning behaviors and AChE activity were examined using a Pearson r correlation coefficient. All data analyses were conducted using SPSS 26 software (IBM, Chicago, USA). ANOVA results and within-dataset correlations were considered significant at *p* < 0.05. Strain AChE mean correlations with Mouse Phenome Database behavioral datasets were considered significant at *p* < 0.01. The significance threshold of *p* < 0.01 was selected as a compromise between statistical lenience and stringency (47) to match the exploratory nature of these analyses.

## Results

### Fear Conditioning

**Figure 1:**
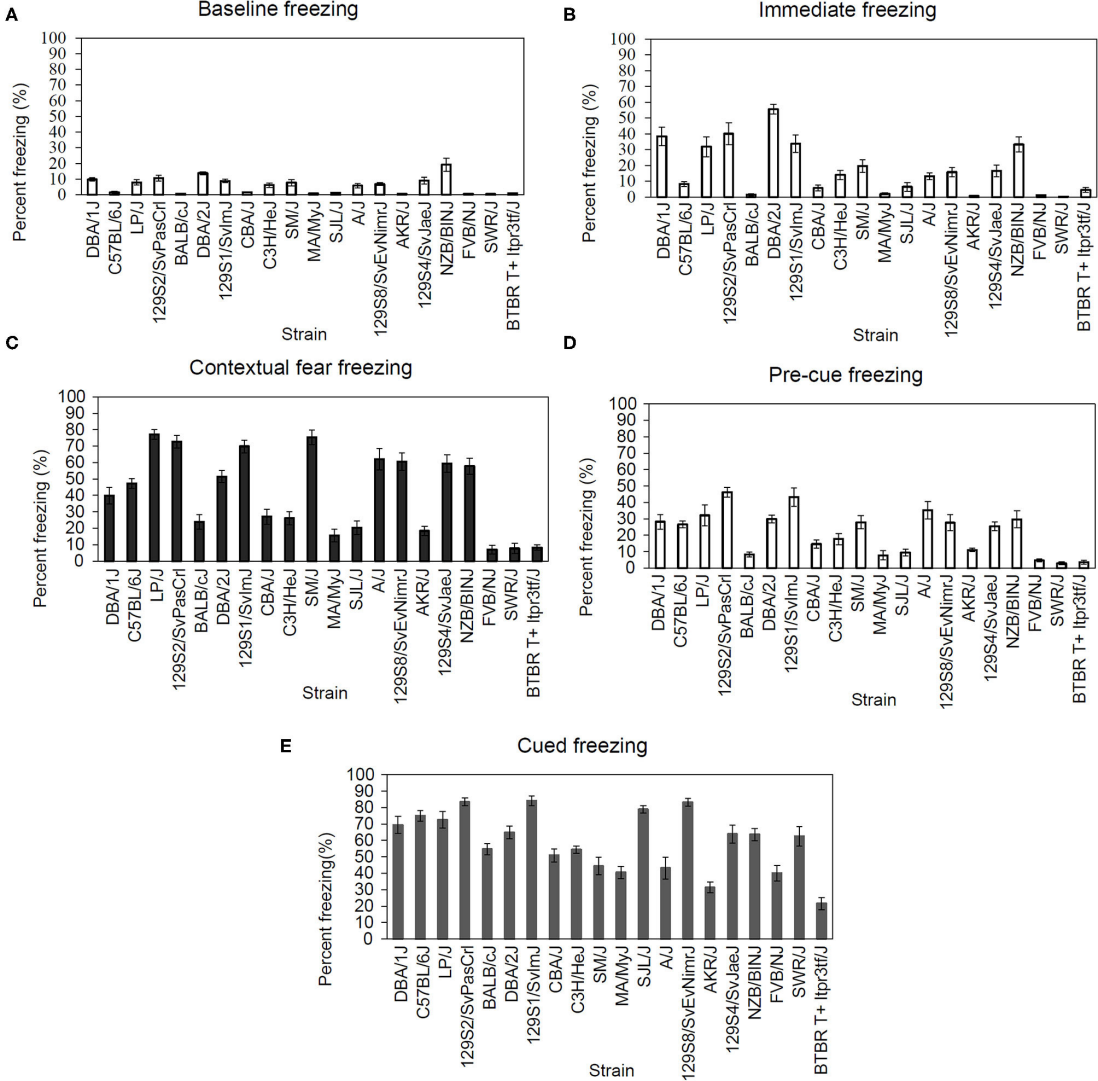
Significant strain differences were present for all fear conditioning components. **(A)** Strain-dependent differences in baseline [*F*_(19,193)_ = 12.81, *p* < 0.05], **(B)** immediate [*F*_(19,193)_ = 20.27, *p* < 0.05], **(C)** contextual [*F*_(19,193)_ = 33.12, *p* < 0.05], **(D)** pre-cue [*F*_(19,193)_ = 13.19, *p* < 0.05], and **(E)** cued [*F*_(19,193)_ = 18.85, *p* < 0.05] freezing during fear conditioning. Data presented as mean ± SEM, *n* = 6–13 per strain. For *post-hoc* comparisons, please see Supplementary Materials.

We examined multiple behavioral components of fear conditioning in 20 inbred strains. The range of freezing responses varied based on the following behaviors: baseline 0.48–19.15%, immediate 0.27–55.61%, context 6.96–77.18%, pre-cue 2.93–46.3%, and cued 21.54–84.14%. The resulting data was analyzed using one-way ANOVAs for the following: freezing during the baseline phase of fear conditioning training [Figure 1A; *F*_(19,193)_ = 12.81, *p* < 0.001], freezing during the immediate (post-shock) phase [Figure 1B; *F*_(19,193)_ = 20.27, *p* < 0.001], freezing to the conditioned context [Figure 1C; *F*_(19,193)_ = 33.12, *p* < 0.001], pre-cue freezing during the cued fear learning test [Figure 1D; *F*_(19,193)_ = 13.19, *p* < 0.001], and freezing to the conditioned cue [Figure 1E; *F*_(19,193)_ = 18.85, *p* < 0.001]. Each ANOVA revealed a significant main effect of strain. The calculated genetic heritability for freezing in each of these stages of fear conditioning were: baseline freezing 57.36%, immediate freezing 67.45%, context freezing 76.32%, pre-cue freezing 58.18%, and cued freezing 66.04%. *Post-hoc* outcomes examining strain differences can be found in Supplementary Tables 4–8.

### AChE Activity

**Figure 2:**
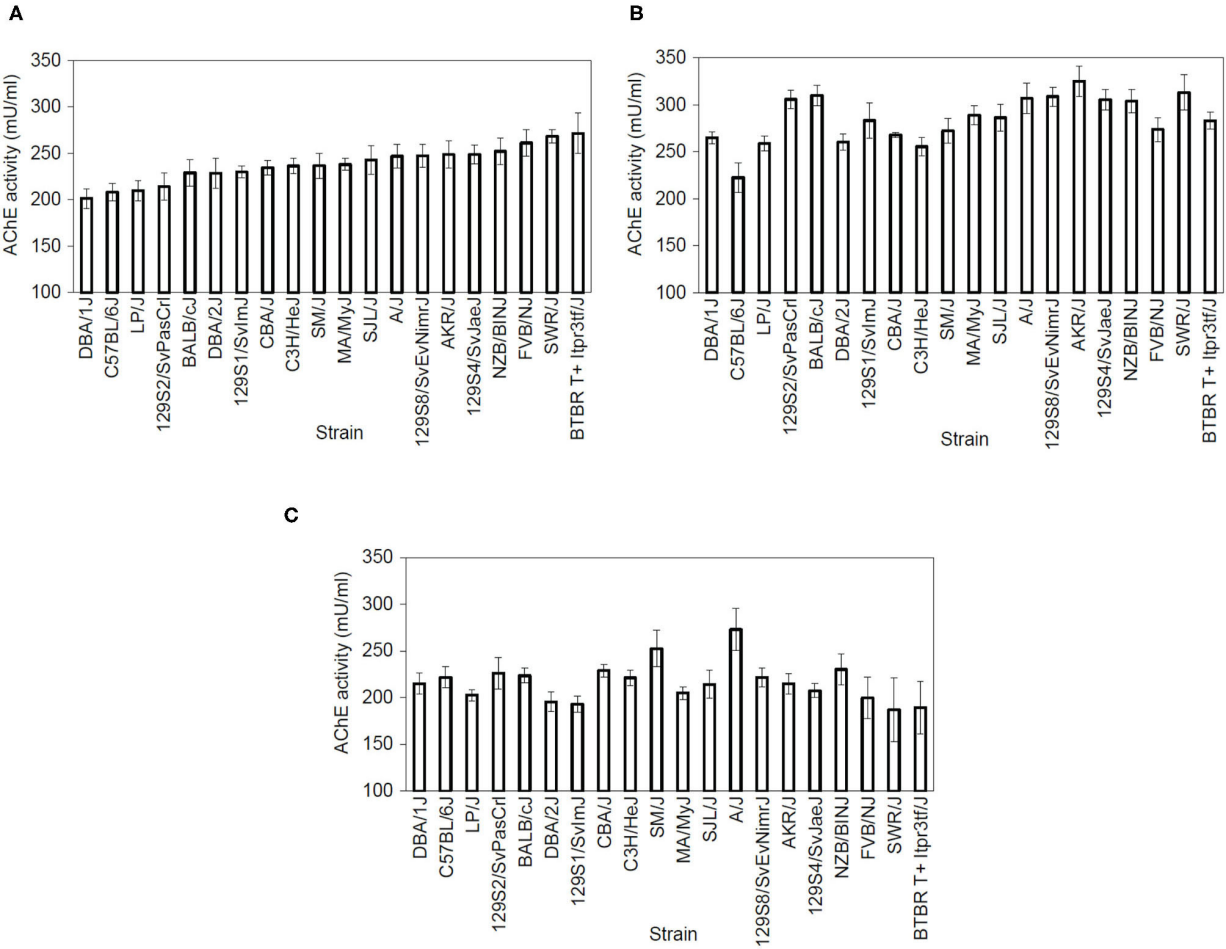
Acetylcholinesterase activity varies by strain. AChE activity (measured in mU/mL) showed a main effect of strain in the **(A)** dorsal hippocampus [*F*_(19,99)_ = 2.37, *p* < 0.05), **(B)** ventral hippocampus [*F*_(19,100)_ = 4.25, *p* < 0.05], and **(C)** cerebellum [*F*_(19,99)_ = 1.81, *p* < 0.05] *n* = 8–11 per strain. Strains are shown from lower to higher AChE activity levels in the dorsal hippocampus and then follow the same order in the ventral hippocampus and cerebellum graphs. For *post-hoc* comparsions, please see Supplementary Materials.

We examined dorsal and ventral hippocampal and cerebellum AChE activity in 20 inbred strains. The range of AChE activity levels varied by region: dorsal hippocampus 201.2–271.3 mU/ml, ventral hippocampus 222.5–325.1 mU/ml, and cerebellum 187–273 mU/ml. The resulting data was analyzed using separate one-way ANOVAs for dorsal hippocampus AChE activity [Figure 2A; *F*_(19,99)_ = 2.37, *p* = 0.003], ventral hippocampus AChE activity [Figure 2B; *F*_(19,100)_ = 4.25, *p* < 0.001] and whole cerebellum AChE activity [Figure 2C; *F*_(19,99)_ = 1.81, *p* = 0.032], which found a significant main effect for strain. Genetic heritability estimates were calculated as follows: dorsal hippocampus AChE activity 34.43%, ventral AChE activity 45.24%, and cerebellum AChE activity 26.15%. *Post-hoc* outcomes examining strain differences can be found in Supplementary Tables 9–11.

### Mouse Phenome Database Gene Polymorphism Queries

To identify genetic variants that could underlie the observed inter-strain differences in AChE activity and learning, we used the Mouse Phenome Database SNP data retrieval tool to examine inter-strain allelic differences in 28 genes related to cholinergic signaling. Their functions and SNPs are listed in Supplementary File 1. Notably, there were 18 polymorphisms within the *AChE* gene, including 5 indels. There were also many polymorphisms in other cholinergic signaling genes.

### Within-Dataset AChE Activity and Fear Conditioning Correlations

To examine potential genetic overlap between behavioral components of fear conditioning, strain mean correlations were calculated between all measured fear conditioning variables (Table 1). Freezing during the baseline of fear conditioning training positively correlated with immediate (post-shock) freezing during training [*r*_(18)_ = 0.87, *p* < 0.001], freezing to the conditioned context [*r*_(18)_ = 0.70, *p* = 0.001], and pre-cue [*r*_(18)_ = 0.73, *p* < 0.001] freezing, but not with freezing to the conditioned cue. Immediate (post-shock) freezing also positively correlated with freezing to context [*r*_(18)_ = 0.69, *p* = 0.001], pre-cue freezing during the cued fear learning test [*r*_(18)_ = 0.79, *p* < 0.001] and freezing to cue [*r*_(18)_ = 0.53, *p* = 0.016]. Context freezing also positively correlated with pre-cue freezing [*r*_(18)_ = 0.93, *p* < 0.001] and cue freezing [*r*_(18)_ = 0.54, *p* = 0.013] freezing. Finally, pre-cue freezing also correlated positively with cue freezing [*r*_(18)_ = 0.60, *p* = 0.005]. These results may point to shared genetic variance underlying freezing during different stages of fear conditioning.

**Table 1:** Pearson correlation coefficients of AChE activity when correlated with different components of fear learning.

	**Baseline**	**Immediate**	**Context**	**Pre-cue**	**Cued**	**DH AChE**	**VH AChE**	**CB AChE**
Baseline	1							
Immediate	0.87^**^	1						
Context	0.70^**^	0.69^**^	1					
Pre-CS	0.73^**^	0.79^**^	0.93^**^	1				
Cued	0.43	0.53^**^	0.54^**^	0.6^**^	1			
DH AChE	−0.23	–0.53^**^	–0.5^**^	–0.58^**^	–0.54^**^	1		
VH AChE	−0.02	−0.22	−0.09	−0.12	−0.14	0.52^**^	1	
CB AChE	0.16	−0.02	0.41	0.35	−0.08	−0.17	0.09	1

* *correlation is significant at 0.05 level*;

** *correlation is significant at the 0.01 level (two-tailed), based on strain means*.

*Bold text represents significant values*.

Strain mean correlations were similarly calculated for AChE activity between brain regions (Table 1). From the three brain regions examined, only a positive correlation between dorsal and ventral hippocampus AChE activity levels was found [*r*_(18)_ = 0.52, *p* = 0.019]. Dorsal and ventral hippocampus AChE activity did not significantly correlate with cerebellum AChE activity. Significant covariance between dorsal and ventral hippocampus AChE activity implies potentially shared genetic factors impacting AChE activity levels between functionally distinct regions of the hippocampus.

**Figure 3:**
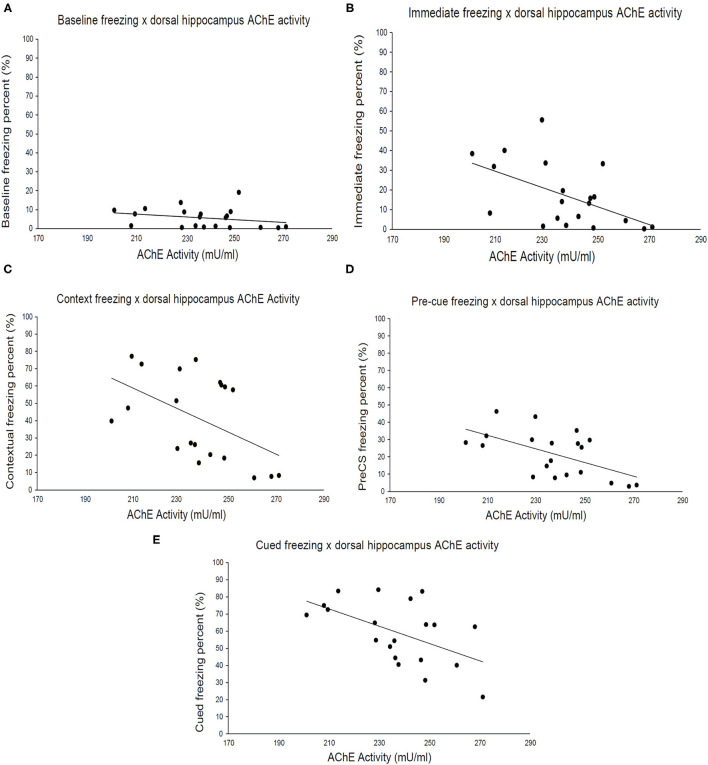
Acetylcholinesterase activity in the dorsal hippocampus correlates with different fear learning components. **(A)** Baseline freezing has a weak correlation (*r* = −0.23, *p* > 0.05) with AChE activity in the dorsal hippocampus, whereas **(B)** immediate (*r* = –*p* < 0.05), **(C)** context (*r* = −0.50, *p* < 0.05), **(D)** pre-cue (*r* = −0.58, *p* < 0.05), and **(E)** cued (*r* = −0.54, *p* < 0.05) freezing negatively correlate with AChE activity. Pearson correlations coefficients are presented in Table 1, *n* = 20 total number of strains.

Finally, we examined the relationship between strain means for brain AChE activity and freezing during fear conditioning. Dorsal hippocampus AChE activity (Figure 3) negatively correlated with immediate (post-shock) freezing during fear conditioning training [Figure 3B; *r*_(18)_ = −0.53, *p* = 0.016], freezing to the conditioned context [Figure 3C; *r*_(18)_ = −0.50, *p* = 0.026], pre-cue freezing during cued test [Figure 3D; *r*_(18)_ = −0.58, *p* = 0.008] and freezing to the conditioned cue [Figure 3E; *r*_(18)_ = −0.54, *p* = 0.015] but not baseline freezing (Figure 3A). No significant correlations between ventral hippocampus or cerebellum AChE activity levels and any of the measured fear conditioning variables were found (Supplementary Figures 1, 2). Thus, genetic factors underlying freezing during multiple stages of fear conditioning covary exclusively with dorsal hippocampus AChE activity, despite significant correlations between ventral and dorsal hippocampus AChE activity.

### Mouse Phenome Database Correlations

Brain AChE activity strain means were correlated with publicly available phenotypes using the Mouse Phenome Database. Only measures with mean data points for a minimum of 8 strains overlapping with our own panel were utilized for MPD correlation analysis. Two thousand twenty-five total measures available in MPD were correlated with AChE activity in each of the tested brain regions (dorsal and ventral hippocampus and cerebellum). We made the *a priori* decision to focus on the top ten significant correlations of AChE with behavioral variables for the purpose of this manuscript.

For dorsal hippocampus AChE activity strain mean correlations, 20 measures met the pre-determined significance cut-off (*p* < 0.01; see Supplementary Table 1). Six of these phenotypes were classified as behavioral models, and three were cognitive assessments in the Barnes maze, as assessed by number of errors that positively correlated with AChE activity. For ventral hippocampus AChE activity (see Supplementary Table 2), 21 measures were considered to be significantly correlated (at *p* < 0.01), with ten of those phenotypes corresponding to a behavioral measure. Specifically, for five out of the six correlated behaviors, AChE activity correlated positively with emotional behavioral responding in cued fear testing, light-dark box, and variants of the elevated maze. Lastly, 30 measures met the significance threshold for correlations with cerebellum AChE activity levels (see Supplementary Table 3). Nine of these corresponded to behavioral measurements. Of note, cerebellum AChE activity correlated positively with six measures of scheduled operant behavior, such as fixed-ratio responding.

## Discussion

Here we report significant variation in fear conditioning and AChE activity in three brain regions across 20 inbred mouse strains. We found that these phenotypes were heritable, especially for freezing during the five stages of fear conditioning assessed (baseline, immediate, context, pre-cue, & cued). Correlation data generated within the current dataset indicate strong positive genetic relationships between freezing levels in various stages of fear conditioning and also between dorsal and ventral hippocampus AChE activity, in addition to a negative correlation between fear conditioning freezing and dorsal hippocampus AChE activity. Correlations of our strain means with publicly available datasets indicate that the dorsal hippocampus AChE activity levels may be more closely related to learning outcomes, whereas ventral hippocampus AChE activity may be better associated with other emotional processing outcomes. Collectively, these findings suggest a degree of heritability in fear learning and hippocampal AChE activity levels and that genetic variability associated with AChE activity in the dorsal hippocampus may contribute to learning in fear conditioning.

### AChE Activity

Using a panel of 20 inbred mouse strains, we found a significant variation of AChE activity in the dorsal hippocampus, ventral hippocampus, and cerebellum. These findings suggest that AChE activity varies by genetic background. Our genetic analysis identified at least 18 polymorphisms in the AChE gene among a subset of the tested strains. Although these polymorphisms did not clearly co-vary with learning or AChE activity in the current panel, they likely contribute in part to inbred strain variation in AChE efficiency and activity. Importantly, enzymatic activity can be modulated by variation in the enzyme itself (e.g., polymorphisms in the encoding gene leading to altered protein structure and changes in enzymatic activity), by distal regulatory genomic elements (e.g., polymorphisms in trans-regulatory elements targeting AChE gene expression), by interactions within the enzymatic pathway (e.g., polymorphisms among broader genomic networks involved in cholinergic signaling), or by a combination of these factors. Our analysis found that related genes encoding choline acetyltransferase (ChAT), an important enzyme in acetylcholine synthesis (48, 49), RIC3, an acetylcholine receptor chaperone protein (50, 51), butyrylcholinesterase, another acetylcholine-metabolizing enzyme (52), and nicotinic acetylcholine receptor subunits (53) also contain numerous polymorphisms across strains. These polymorphisms could all potentially influence cholinergic signaling and represent possible mechanisms through which genetic differences across strains influence AChE activity and learning. Moreover, the large amount of cholinergic modulating genes exhibiting polymorphisms suggests that the observed phenotypic differences in learning may involve complex genotypic and regulatory interactions.

### Fear Conditioning

The current results are in line with previous findings of genetic variability in conditioned fear learning (37, 54, 55). Analyses of our inbred mouse strain panel indicated significant between-strain variation in freezing during multiple stages of fear conditioning (baseline, immediate, context, pre-cue, & cued). Moreover, these behaviors were highly heritable, all demonstrating >57% heritability. Our fear conditioning paradigm measured two distinct associations: First, between the training context (contextual fear conditioning) and the US, and second, between the CS and US (cued fear conditioning). Here, we report a wide range of contextual fear conditioning across our inbred strain panel with some strains demonstrating high (LP/J, SM/J, 129S2, & 129S1, >68% freezing) and some demonstrating low contextual fear conditioning (FVB/NJ, SWR/J, & BTBRT +, <10% freezing). The differences in the mice that show high levels of freezing and the mice that show low levels of freezing during the context tests could suggest that the high and lower responders differ in hippocampus-dependent learning. In support, studies examining LP/J, SM/J, & 129S1 inbred mice have found high levels of learning in hippocampus-dependent tasks (56) including contextual fear conditioning (57, 58) relative to other tested strains. Similarly, FVB/NJ, SWR/J, and BTBRT+ strains exhibit low levels of hippocampus-dependent learning (35, 59–61). It should be noted that FVB/NJ and SWR/J strains possess a *Pde6b* gene mutation that leads to compromised visual acuity (62). However, work conducted by Bolivar et al. (36) indicated that retinal degeneration produced by this mutation did not impact contextual fear learning. Moreover, the C3H/HeJ strain shares a similar *Pde6b* mutation but displayed >20% freezing, suggesting that factors outside of visual acuity contributed to the observed strain differences. Interestingly, the three strains with the lowest contextual fear learning also had the highest levels of dorsal hippocampus AChE activity. Low contextual fear conditioning in these strains may be explained in part by higher dorsal hippocampus AChE activity levels (discussed more below) as acetylcholine is critically involved in learning (63) but other differences could also contribute to the differences in fear conditioning.

In addition to contextual fear learning, we found significant between-strain variation in cued fear conditioning. Our results indicate that the BTBRT+ strain exhibits poor cued fear conditioning (~21% freezing across trial), which is supported by other studies (64). BTBRT+ strain displays abnormal amygdala nuclei volume (65). Given the role of the amygdala in cued fear conditioning (39), it is possible that structural abnormalities within this region may account for the deficits reported here.

### Within-Dataset and Mouse Phenome Database Correlations

Strain mean correlations are useful for identifying potential relationships between the genetic influence of behavioral and biological outcomes. Here, we found that dorsal hippocampus AChE activity was significantly negatively correlated with freezing in all stages of fear conditioning except for baseline freezing. This finding suggests that genes that influence dorsal hippocampus AChE activity may contribute to variability in fear conditioning but not baseline levels of activity as assessed by freezing. Dong et al. (66) reported significant improvement in contextual fear conditioning in a mouse model of Alzheimer's disease following injections of AChE inhibitors physostigmine or donepezil. Moreover, nicotine withdrawal- and MK-801-induced deficits in contextual fear conditioning were prevented after administration of an AChE inhibitor (67–69). Notably, Csernansky et al. (67) observed inconsistent improvement in contextual fear conditioning via inhibition of AChE in saline-treated mice, suggesting that improvement in contextual fear conditioning via inhibition of AChE may be dependent on altered cholinergic signaling. The genetic association between dorsal hippocampus AChE activity and learning was further supported by our MPD analysis, which found that dorsal AChE activity strain means were positively correlated with the number of errors committed during the Barnes maze (see Supplementary Table 1), a hippocampus-dependent learning task. Qualitative support for this idea comes from the fact that the bottom three strains with the lowest freezing to the conditioned context (SWR/J, BTBRT+, and FVB/NJ) displayed the highest dorsal hippocampus AChE activity, indicating that enhanced AChE activity may impair contextual fear learning. Alternatively, it is possible that genetic differences in genes associated with AChE reflect or contribute to systemic alterations in cholinergic function, which is critical for learning (70, 71).

Both ventral hippocampus and cerebellum AChE activity failed to correlate with fear conditioning variables. Previous reports suggest that the dorsal and ventral hippocampus carry out distinct behavioral functions despite being a continuous anatomical structure (41). For instance, the dorsal hippocampus plays a role in spatial learning and memory (72, 73), whereas the ventral hippocampus is involved in emotional processing and stress responding (74). Although we did not examine more traditional behavioral paradigms of emotional processing (e.g., elevated plus maze or light dark box), strain mean correlations with external datasets in MPD indicated significant associations between ventral hippocampus AChE activity and behavioral variables potentially representing emotional processing (see Supplementary Table 2). Interestingly, higher AChE activity in the ventral hippocampus was associated with greater frequency of urination and fecal boli in the light-dark box and elevated plus-maze. This suggests that increased AChE activity may predict greater levels of anxiety. It is worth noting that fear learning has emotional components and manipulation of ventral hippocampus functioning can influence fear learning (75). Moreover, recent findings from Giacomini et al. (76) suggest that inhibition of AChE via donepezil increases anxiety-like behavior in zebrafish in a dose-dependent manner. It is feasible that overlapping genes mediate ventral hippocampus AChE activity and anxiety-like behavior. However, the relationship between anxiety and AChE activity in the ventral hippocampus cannot be reliably surmised from the current data as there was no significant correlation with fear conditioning. Traditional measures of anxiety would need to be assessed to see if strain variability in the ventral hippocampus contributed to altered anxiety phenotypes.

### Limitations/Conclusions

The current study's goal was to examine genetic variation in fear conditioning, as well as AChE activity in the hippocampus and cerebellum. Our findings indicate that all five components of fear conditioning and AChE activity in three brain regions (dorsal and ventral hippocampus and cerebellum) significantly differed based on genetic background. Additionally, strain means correlational analysis found a negative relationship between the dorsal hippocampus AChE activity and fear conditioning, suggesting genetic variability in AChE activity contributes to differences in learning. While these findings have interesting implications for the role of AChE-related genetics in learning it is important to highlight their correlational nature. That is, the current study does not provide causal evidence that AChE differences may or may not be due to genetic differences. Future studies can also examine cholinergic markers in regions such as the amygdala, which is also importantly involved in fear conditioning (77). Additionally, correlations with publicly available datasets are limited by the number of overlapping strains. Moreover, it must be noted that all animals in the current study underwent surgical procedures, including osmotic minipump implantation (saline). Although animals were not subjected to drug exposure, it is possible that surgical stress may have influenced measured outcomes. Lastly, the scope of the current study is limited by using males only.

Collectively, our data provide further evidence that biological and behavioral outcomes are influenced by genetic background. Additionally, fear conditioning and dorsal hippocampus cholinergic signaling appear to be associated and mediated by common genetic factors. Additional research may help elucidate potential genetic targets influencing fear conditioning and AChE activity, as well as mechanisms possibly mediating the relationship between dorsal hippocampus AChE activity and fear conditioning.

## Data Availability Statement

The original contributions presented in the study are included in the article/Supplementary Material, further inquiries can be directed to the corresponding author/s.

## Ethics Statement

The animal study was reviewed and approved by the Pennsylvania State University Institutional Animal and Care Use Committee.

## Author Contributions

SM-L and DZ: conceptualization, data collection, analysis, and writing. PG-T and LS: data collection, analysis, and writing. MB: analysis and editing. SG: analysis. GP: funding and editing. TG: conceptualization, writing, editing, and funding. All authors contributed to the article and approved the submitted version.

## Funding

This study was supported by the National Institutes of Health [T32GM108563 (LS), U01DA041632 (TG), F31DA049395 (DZ), and U01DA044399 (GP)], the Jean Phillips Shibley Endowment (TG), and Penn State University.

## Conflict of Interest

The authors declare that the research was conducted in the absence of any commercial or financial relationships that could be construed as a potential conflict of interest.

## Publisher's Note

All claims expressed in this article are solely those of the authors and do not necessarily represent those of their affiliated organizations, or those of the publisher, the editors and the reviewers. Any product that may be evaluated in this article, or claim that may be made by its manufacturer, is not guaranteed or endorsed by the publisher.
