# Supplementary figures and images for: Genetic Differences in Dorsal Hippocampus Acetylcholinesterase Activity Predict Contextual Fear Learning Across Inbred Mouse Strains
Source: Front Psychiatry. 2021 Oct 18;12:737897. doi: 10.3389/fpsyt.2021.737897 (PMC8558262; doi:10.3389/fpsyt.2021.737897)

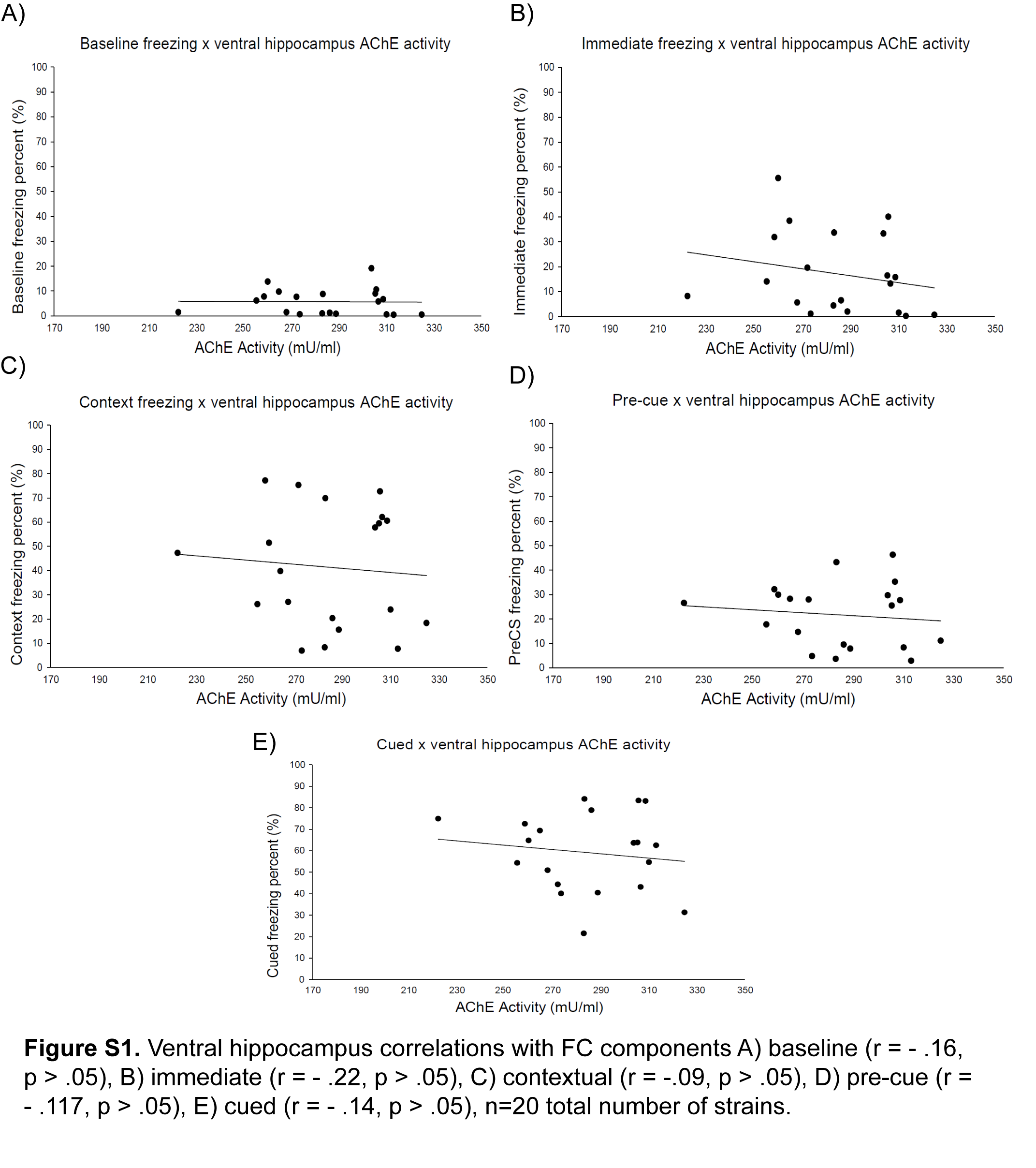

Supplement: Supplementary file 1 [file Image_1.TIF]

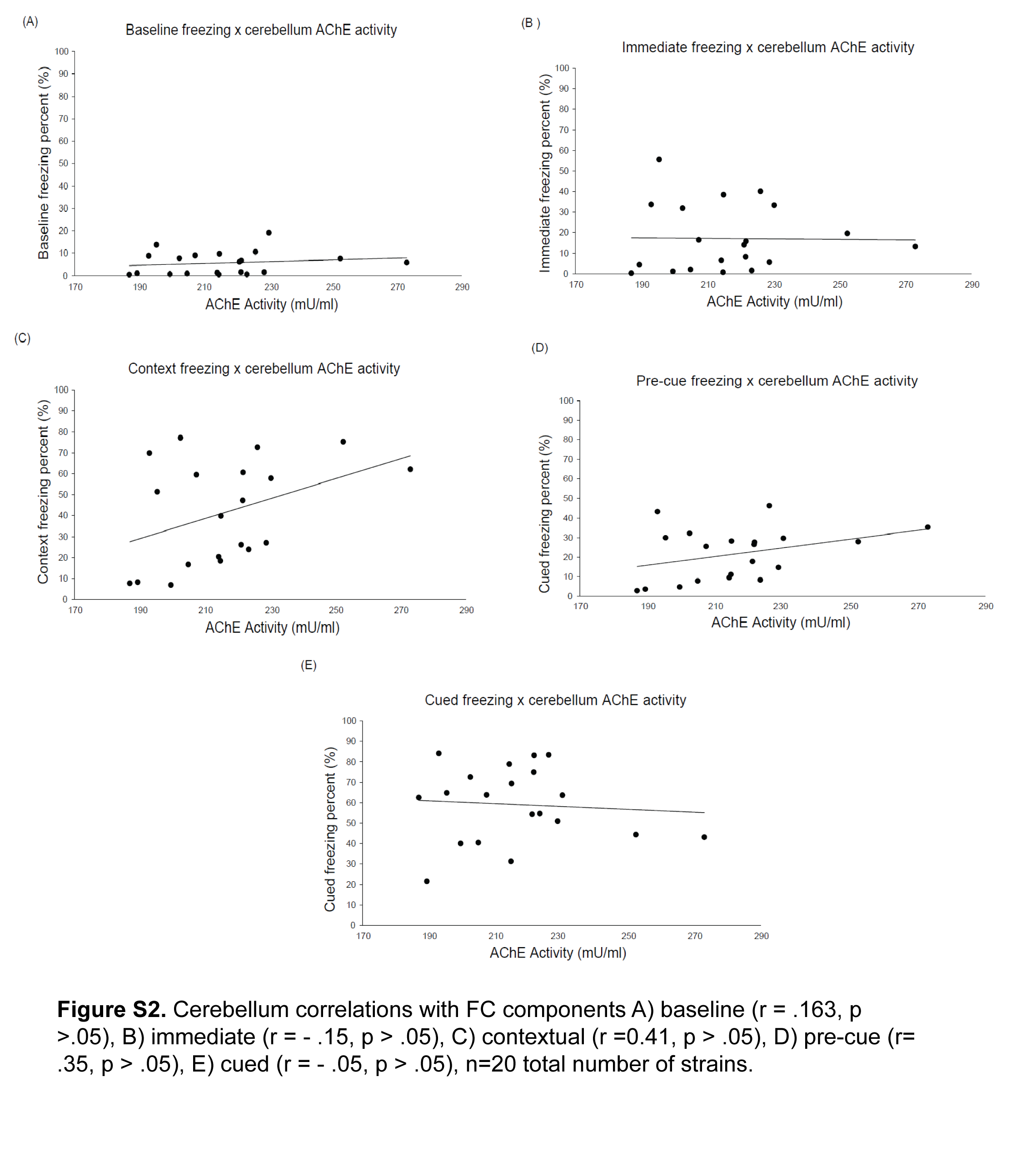

Supplement: Supplementary file 2 [file Image_2.TIF]

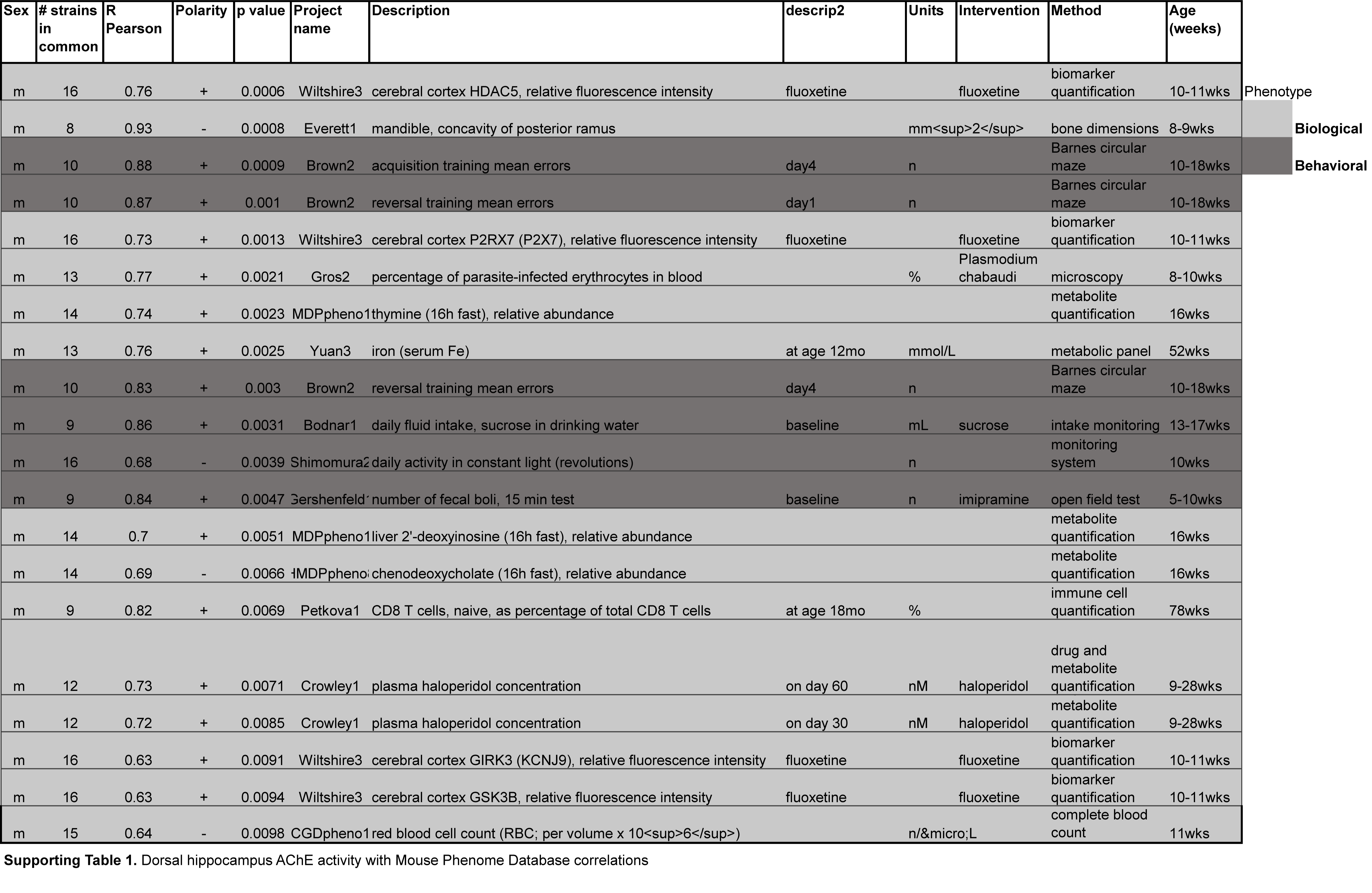

Supplement: Supplementary file 3 [file Image_3.TIF]

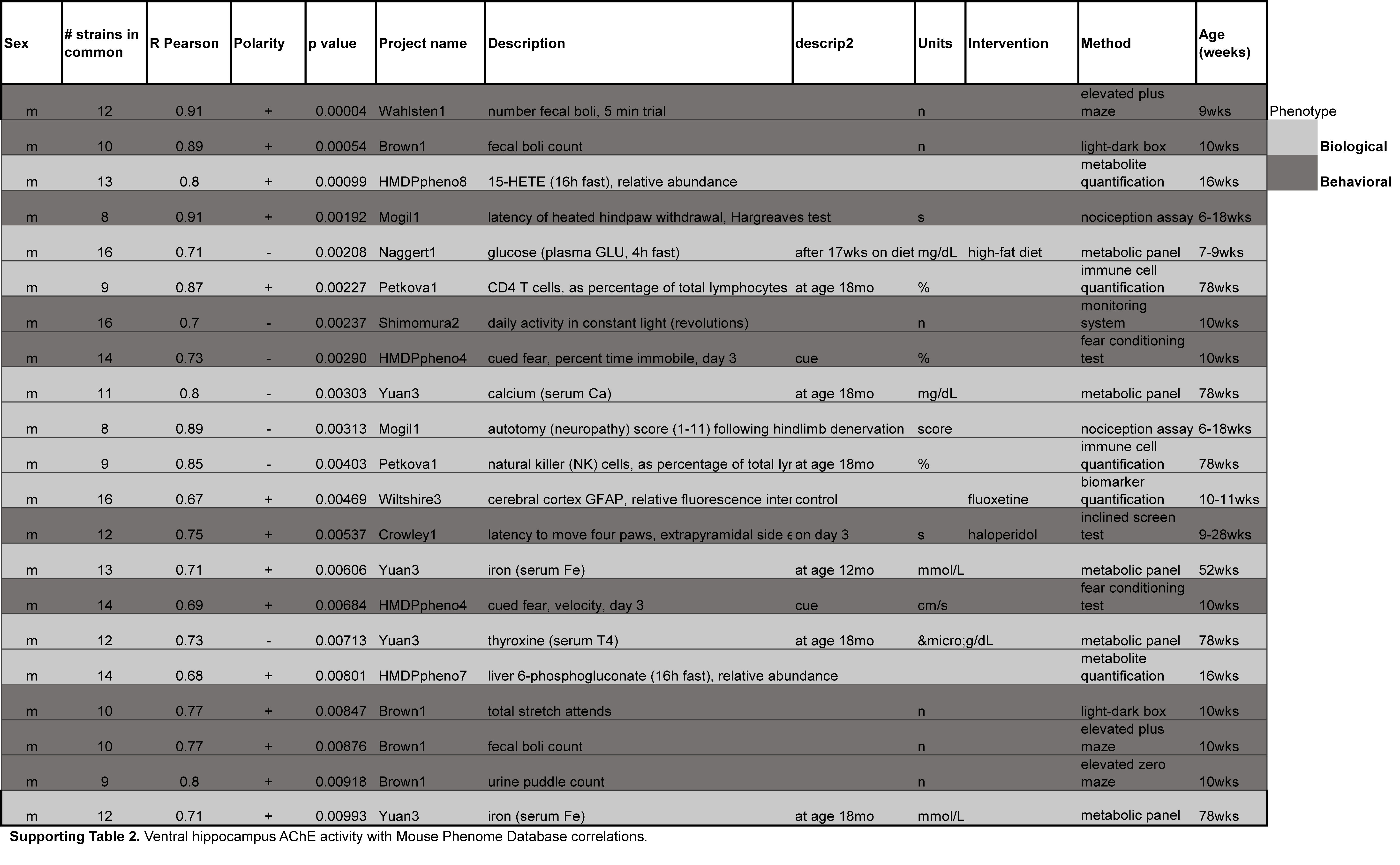

Supplement: Supplementary file 4 [file Image_4.TIF]

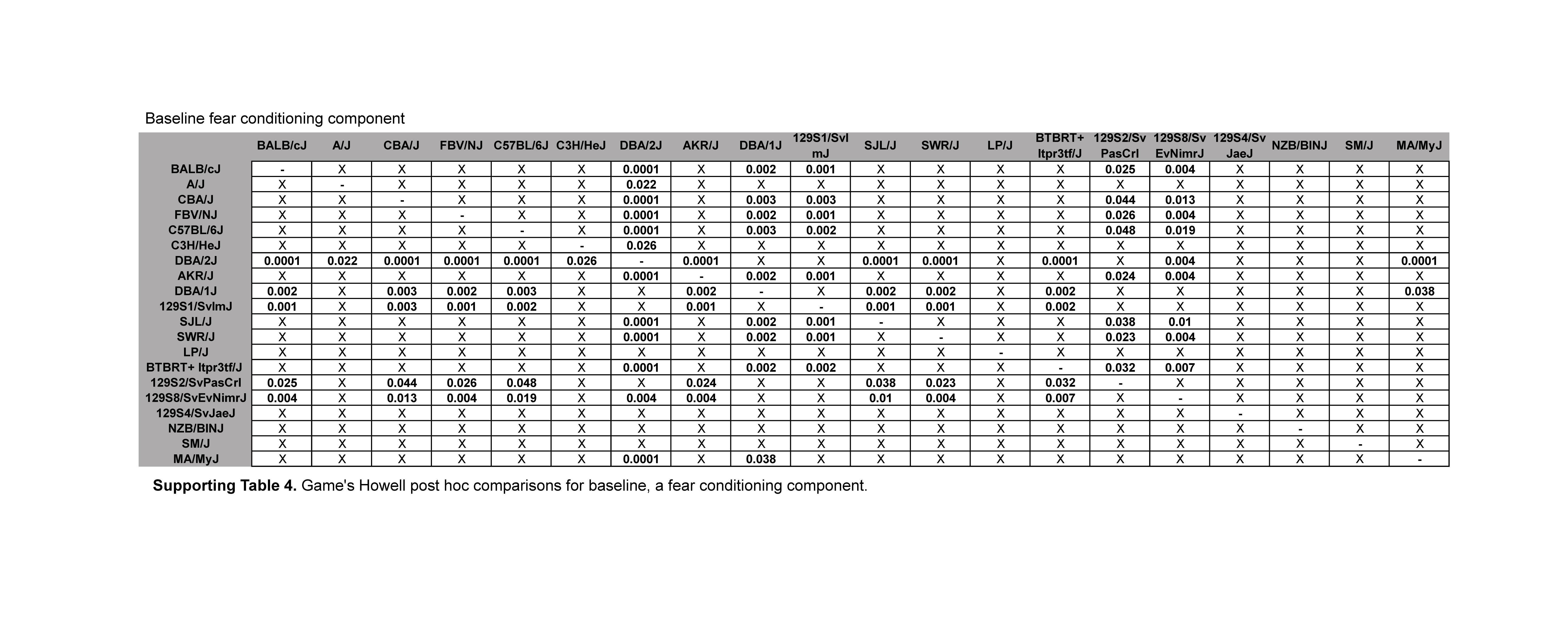

Supplement: Supplementary file 6 [file Image_6.TIF]

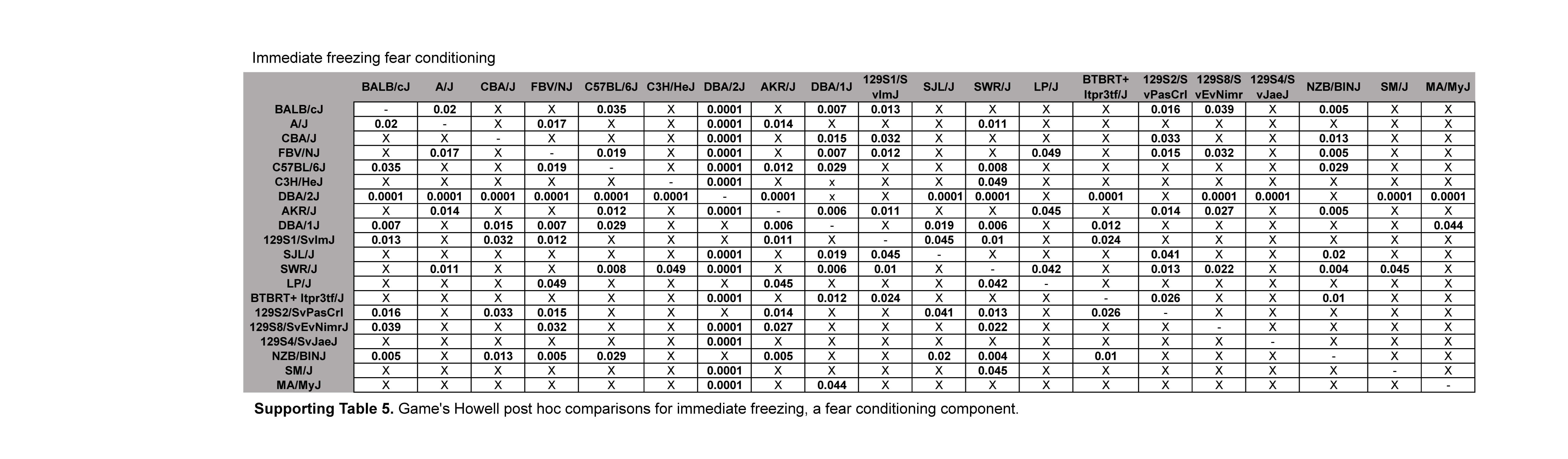

Supplement: Supplementary file 7 [file Image_7.TIF]

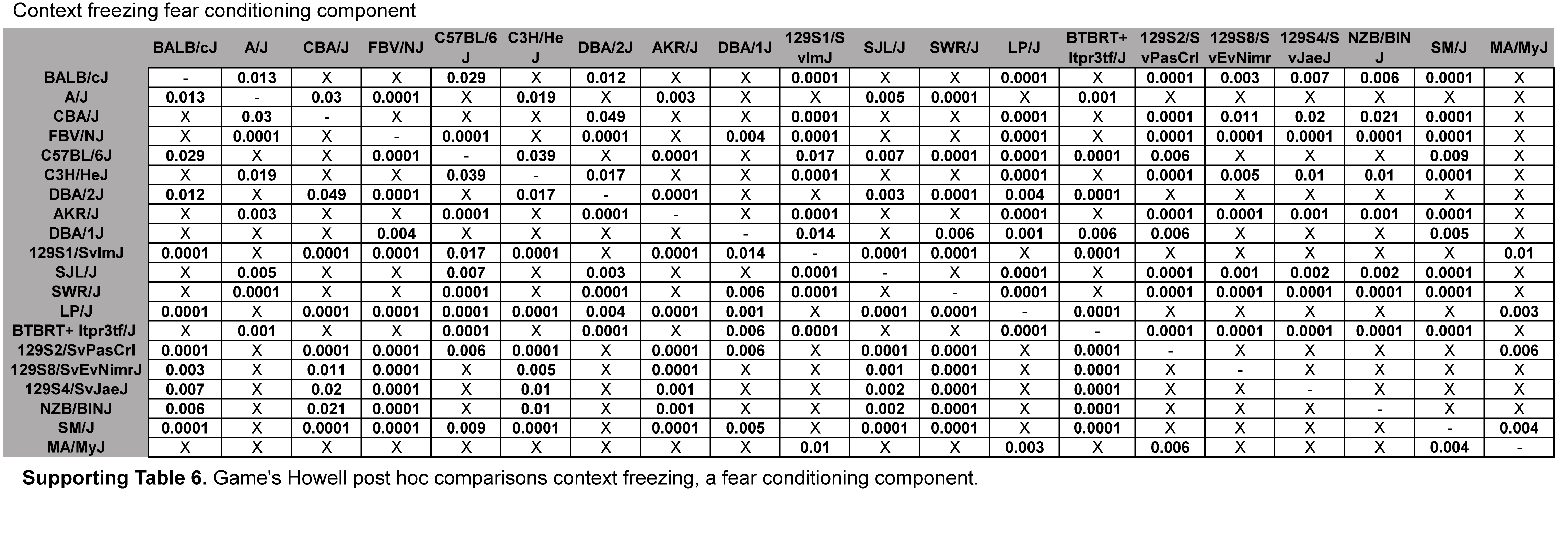

Supplement: Supplementary file 8 [file Image_8.TIF]

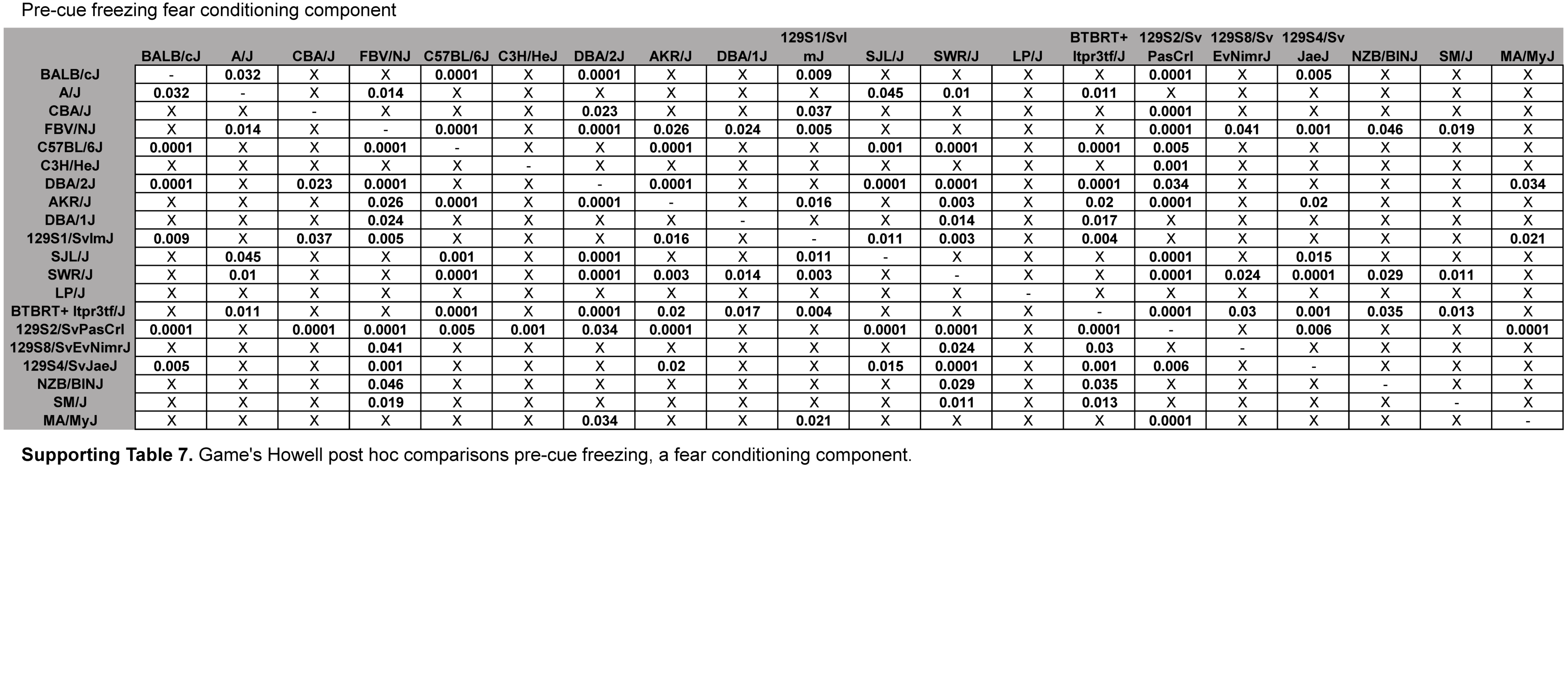

Supplement: Supplementary file 9 [file Image_9.TIF]

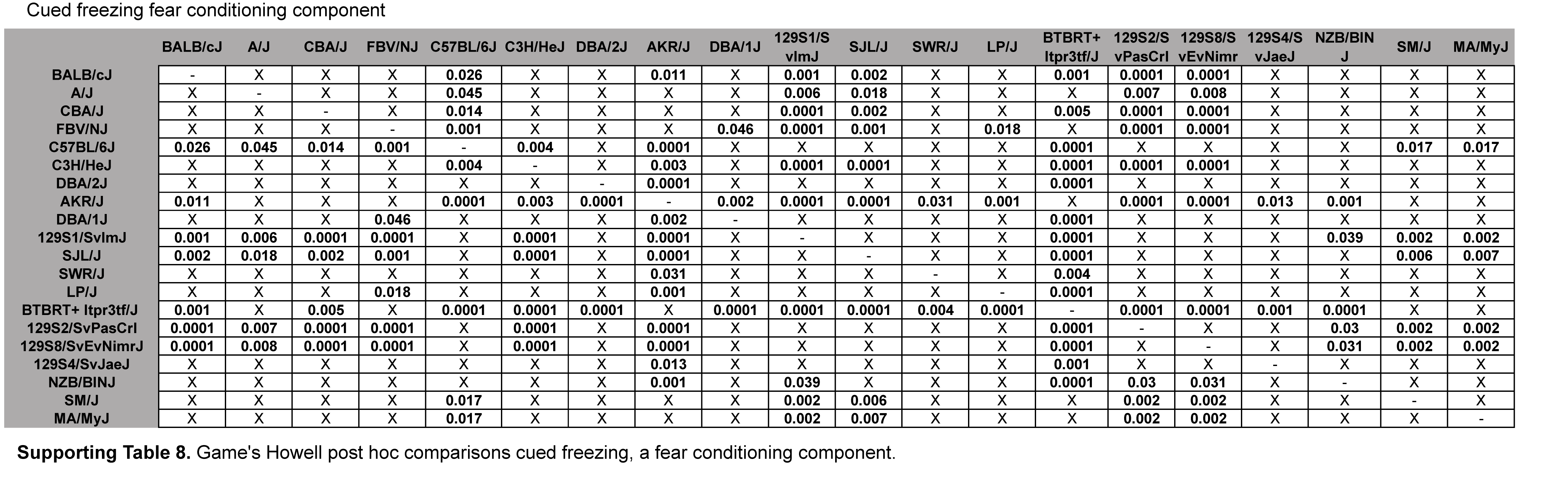

Supplement: Supplementary file 10 [file Image_10.TIF]

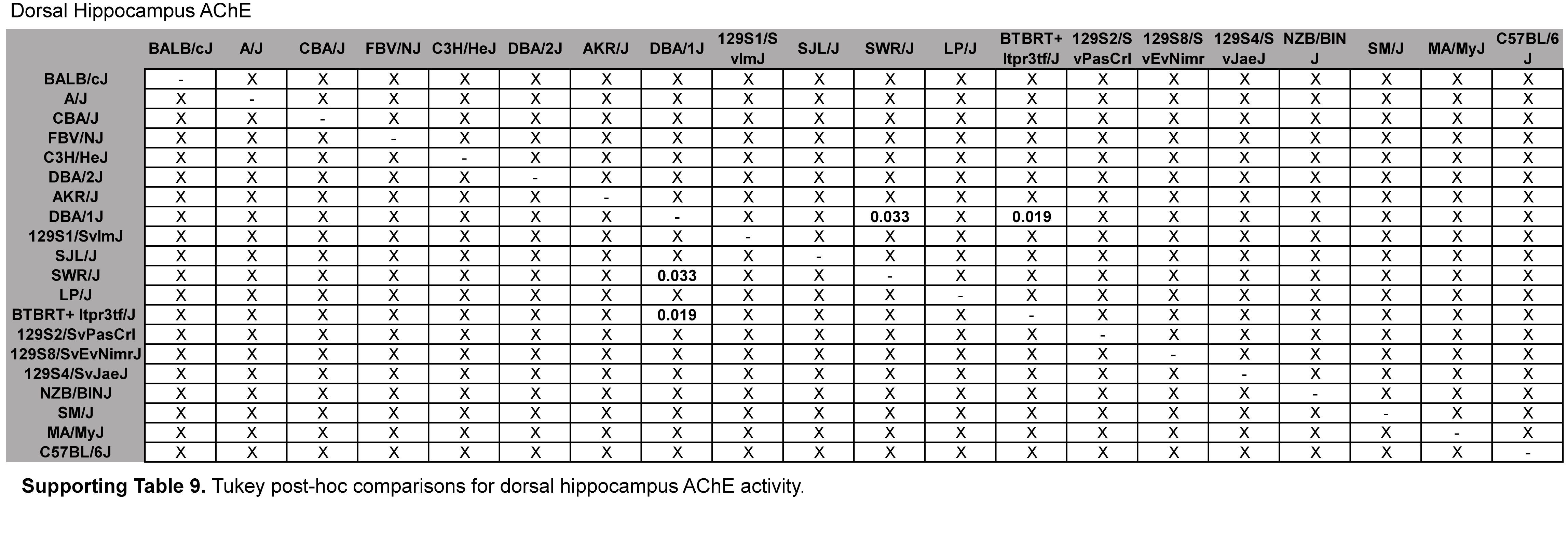

Supplement: Supplementary file 11 [file Image_11.TIF]

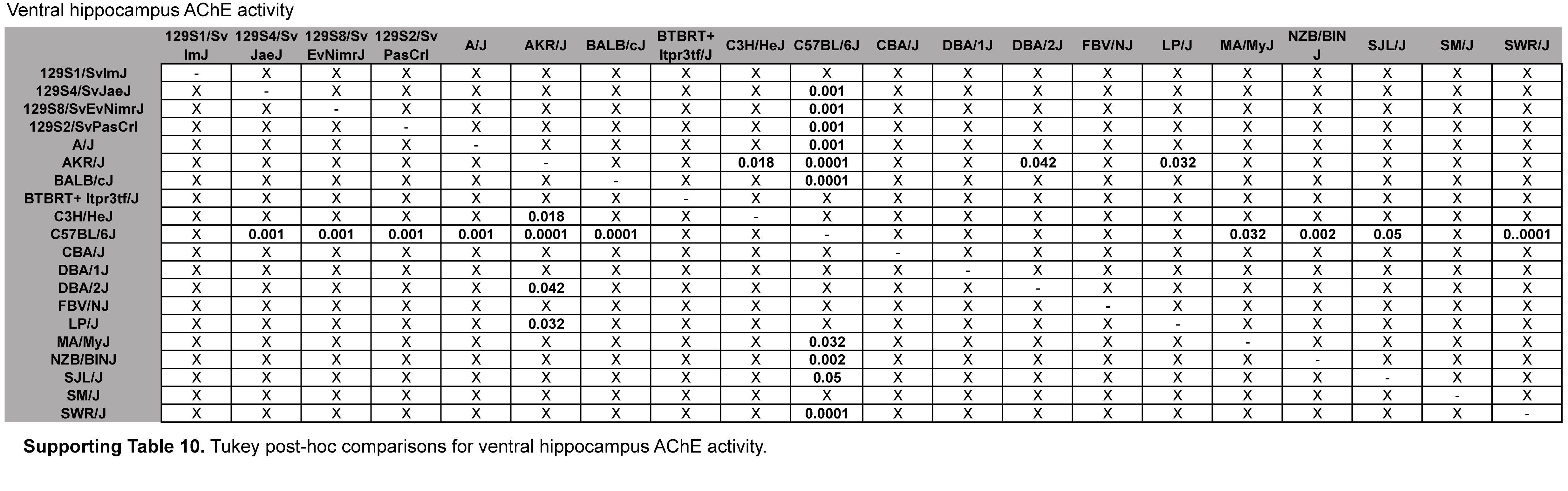

Supplement: Supplementary file 12 [file Image_12.TIF]

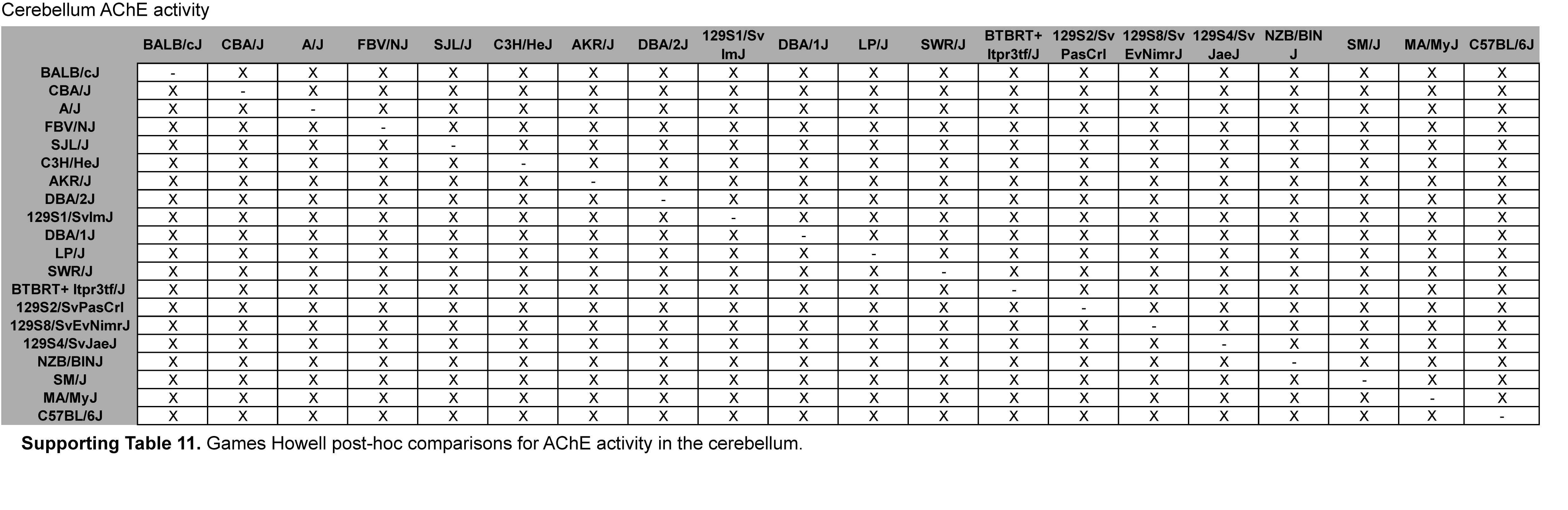

Supplement: Supplementary file 13 [file Image_13.TIF]
